# Personal

**Published:** 1905-02

**Authors:** 


### PERSONAL.

Dr. Marshall Clinton, of Buffalo, has been commissioned assistant surgeon of the 65th Regiment N. G. N. Y., vice Dr. F. C. Busch resigned. This appointment carries with it the rank of captain, and Dr. Clinton comes into the service with ample experience, having served as assistant surgeon of the 202d N. Y. volunteers during the Spanish war.

Dr. Charles Sherman Jewett, of Buffalo, was elected president of the medical staff of the Sisters of Charity Hospital at the last annual meeting of the attending physicians and surgeons, who constitute that body.

Dr. Benjamin H. Grove, of Buffalo, was elected president, Dr. Francis J. Carr, vice-president, and Dr. Vertner Kenerson, secretary, of the Emergency Hospital at the recent annual meeting of the medical staff.

Dr. Roswell Park, of Buffalo, delivered an address on the History of surgery in America at a meeting of the Buffalo Historical Society, held January 8, 1905.

Dr. L. S. McMurtry, of Louisville, president of the American Medical Association, recently spent a day in Buffalo, the guest of Dr. William Warren Potter. Dr. McMurtry was on his way to Albany, to attend, by invitation, the ninety-ninth annual meeting of the Medical Society of the State of New York.

---

Dr. D. W. Harrington, of Buffalo, who was quite ill in December, has become convalescent and is journeying in the south to complete his recovery.

---

Dr. George F. Cott, of Buffalo, announces the removal of his office to No. 85 North Pearl street, in the Dr. Bennett block. Bell telephone.

---

Dr. A. H. Briggs, of Buffalo, who has served twenty-five years in the medical corps of the 65th Regiment N. G. N. Y., rising to the rank of major-surgeon, was recently promoted to be lieutenant-colonel by brevet for faithful and meritorious service. Colonel Briggs was also the recipient, a few weeks ago, of a testimonial dinner at which the hospital corps of his regiment presented him with a sword in token of respect and esteem, and in commemoration of the twenty-fifth anniversary of his military service. Seldom do such marked honors fall to a medical officer in the state national guard service, and none could be more deserving of such than our distinguished colleague, Colonel Briggs.

Dr. Briggs was elected vice-president of the Medical Society of the County of Erie during its annual meeting held at Buffalo, January 10, 1905.

---

Dr. James L. Gallagher, of Buffalo, was recently appointed visiting physician at the Emergency Hospital, vice Dr. John H. Pryor resigned.

---

Dr. John H. Pryor, formerly of Buffalo, now superintendent of the New York State Hospital for Incipient Tuberculosis at Raybrook, visited his old home a few days ago, receiving a cordial welcome from his many friends in this city.

---

Dr. Edwin Walker, of Evansville, Ind., has recently completed a seven months' sojourn in Europe, most of which time was spent in the surgical clinics of the old world. Dr. Walker is at the head of one of the largest and most successfully conducted sanitariums in the mid-southwest, and resumes his large practice splendidly equipped to deal with the intricate questions that confront him.

